# Pigs’ management practices and exposure to *Trichinella* spp. in pigs and warthogs in the northern area of Senegal

**DOI:** 10.14202/vetworld.2022.2253-2258

**Published:** 2022-09-20

**Authors:** Kacou Martial N’da, Oubri Bassa Gbati, Laibané Dieudonné Dahourou, N’guessan Ezéchiel Schadrac Behou, Amadou Traore, Joseph Kungu

**Affiliations:** 1 Parasitology and Mycology Laboratory, Public Health - Environment Department, Inter-State School of Veterinary Science and Medicine - EISMV, Dakar, Senegal; 2 Department of Livestock Breeding, Rural Development and Environmental Sciences Institute (ISEDR), University of Dedougou, Dedougou, Burkina Faso; 3 Laboratory of Animal Health and Biology, Department of Animal Productions, Environment and Agricultural Research Institute (INERA), Ouagadougou, Burkina Faso; 4 Department of Biosecurity Ecosystems and Veterinary Public Health, College of Veterinary Medicine Animal Resources and Biosecurity, Makerere University, Kampala, Uganda

**Keywords:** epidemiological studies, pig management, Senegal, *Trichinella* infection, warthog

## Abstract

**Background and Aim:**

Trichinellosis is a neglected and emerging foodborne zoonosis in Africa. *Trichinella* infection occurs through the consumption of raw or undercooked infected meat and meat products. This study aimed to assess pigs’ management practices and determine the exposure of pigs and warthogs to *Trichinella* spp. in the northern area of Senegal.

**Materials and Methods:**

Surveys and observations were carried out among 40 pig farmers to assess husbandry practices regarding *Trichinella* spp. life cycle. In addition, 201 pig meat juices and 83 warthog meat juices were extracted and tested for anti-*Trichinella* antibodies by indirect enzyme-linked immunosorbent assay.

**Results:**

Most (97%) of farms practiced a traditional farming system with free-ranging of pigs in 85% of farms. Farms had local pig breed without housing and supplementary feeding. Some farmers (27.5%) used slaughter waste to feed pigs and farmers were not aware that free-range farming is a source of infection to *Trichinella* infection. They were also unaware that some pig diseases could be transmitted to humans. The seroprevalence of *Trichinella* infection was 10.9% (95% confidence interval [CI]: 6.6–15.2%) in pigs and 10.8% (95% CI: 4.16–17.52%) in warthogs with significantly higher seroprevalence in male (22.2%: 95% CI: 6.6–37.8%) compared to female (9.2%; 95% CI: 4.9–13.5%) (p *<* 0.05).

**Conclusion:**

The confirmation of exposure to *Trichinella* spp. in this area in pigs and warthogs shows a significant risk of transmission of this disease to humans if the farming conditions and the health surveillance system are not respected. However, control measures are needed to reduce any risk of transmission of *Trichinella* infection to humans.

## Introduction

Foodborne zoonoses are a threat to public health as more than 420,000 deaths are recorded worldwide each year [1] and most cases are noted in African and South Asian countries. *Trichinella* infection is one of the major public health problems worldwide. It affects more than 150 species of mammals, birds, and reptiles and is listed as an animal disease by the World Organization for Animal Health [2]. Of the species affected, wild carnivores and domestic animals such as pigs are mainly involved in the life cycle of the parasite. Transmission occurs through the consumption of raw or undercooked meat or meat products infected with *Trichinella* spp. [3]. The disease is asymptomatic in animals but can cause symptomatic disease in humans [4]. In Senegal, trichinellosis was first documented following the consumption of warthog meat by nine European travelers [5, 6]. In 2009, three cases were also reported involving three tourists who consumed smoked warthog ham in Saint-Louis [7]. Despite the presence of the disease, inspection of pork and warthog meat is not systematic.

In the Saint-Louis region, warthog meat intended for human consumption is not inspected and is sold directly for human consumption. With the omnivorous behavior of warthogs [8], they can be infected by *Trichinella*. As for pork, inspection is still insufficient because there are uncontrolled marketing channels that do not respect any measures in this region. Despite the development of the pig industry, the traditional farming system (a farming system where animals is ranging free, without housing, supplementary feeding, and with less medical care) dominates in this region. Free-ranging pig husbandry could be a risk factor for *Trichinella* infection as during their free-roaming, pig could ingest infected rats [9]. Pigs and warthogs could be infected by *Trichinella* because of their omnivorous behavior. However, no studies have been conducted to describe the husbandry practices and exposure to *Trichinella* in pigs and warthogs in this region, which is the second largest supply site for warthogs in Senegal.

This study aimed to assess pigs’ management practices and determine the exposure of pigs and warthogs to *Trichinella* spp. in the northern area of Senegal.

## Materials and Methods

### Ethical approval and Informed consent

This study was non-invasive as it consisted of sampling meat for consumption to detect anti-*Trichinella* antibodies in the meat juices that were extracted from these muscle samples. Oral consents were obtained from farmers before the implementation of interviews.

### Study period and location

The study was conducted from December 2020 to July 2021. The Saint-Louis region covers an area of 19,034 km², about 10% of Senegal’s surface area, with 6.6% (1.036.009 inhabitants) of the national population in 2018. The region comprises three divisions: Saint-Louis, Dagana, and Podor. The region’s climate is Sahelian. The national pig population was estimated at 423,000 head in 2016, an increase of 3.7% compared to 2015 [10]. The region is known to be an economically important pig farming area for Senegal. Traditional and semi-intensive systems are the most represented farming systems in this region. The pig samples were obtained mainly from the localities of Richard Toll, Ross-Bethio, Saint-Louis, and Savoigne. However, the region is rich in faunal diversity, which has led to the creation of hunting areas for warthogs. This abundance could often lead to promiscuity between stray pigs and warthogs, especially in the localities of Richard Toll. In addition to the above-mentioned localities, warthog harvests also came from Djama and Djoudj National Park.

### Sampling strategy and determination of the meat sample size

The sampling of farmers in the Saint-Louis region was based on the list of pig farmers obtained from the veterinary services in that region. We used the exhaustive sampling formula [11] to determine the number of pig farms (n) to be surveyed from a list of 93 farmers (N) provided by the veterinary services:


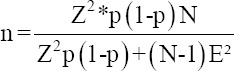


Where, n = sample size; p = expected proportion of farmers who are aware of *Trichinella* infection (set at 50%); E = precision (set at 10%); Z = value on the Z table corresponding to a confidence threshold (90%) = 1.645; and N = size of the whole population. After applying the formula in total, the minimal sample size for this study was 40. For the detection of exposure to *Trichinella*, pork and warthog meat samples were collected. The sample size of pork and warthog meat was calculated according to Trusfield [12] as follows: n =Z ² *p (1–p)/m² with an expected prevalence of 4% [5] in a study conducted on warthogs between Mauritania and Senegal. In this formula, n is the required sample size; Z= a value on the z table corresponding to a 95% confidence level (1.96); p is the expected prevalence (4%); and m is the precision (5%). Using these data, the minimum sample size was 60 for each species, but 201 pork samples and 83 warthog samples were collected for this study. For the selection of pork samples, a list of pork sellers in the region was obtained from the veterinary services. A visit was then made to each meat selling area to collect samples every 3 days during the study period. Warthog meat sampling was carried out during the same period. For this sampling, warthog hunters and meat sellers were identified in the study area and meat samples were collected according to the availability of warthog meat.

### Surveys, meat sampling, and juice collection

Data were collected in two stages. First, interviews were conducted with pig farmers using a semi-structured questionnaire. Data on socioprofessional information, knowledge about *Trichinella* infection, practices, and management methods on the farms related to the life cycle of *Trichinella* were collected. In addition, for pork and warthog samples, 50–80 g samples of pork or warthog meat were collected and packed in sterile bags. All samples were kept in a freezer at −20°C until the juices were collected in the laboratory. This was done by thawing each sample to extract the maximum amount of juice. The extracted juices were stored in Eppendorf tubes and kept at –20°C until enzyme-linked immunosorbent assay (ELISA) analysis.

### Laboratory analysis of samples

The laboratory analysis was carried out using the ID Screen^®^ *Trichinella* Multi-species test – (IDvet, France). This indirect multispecies ELISA kit is used to detect anti-*Trichinella* antibodies in the meat juices of Suidae. The test uses the *Trichinella* excretory/secretory products which are added to each well of the ELISA plate. Test samples and controls were filled into the wells. If *Trichinella* antibodies are present, they constitute an antigen-antibody complex. After washing, a peroxidase multispecies conjugate (horseradish peroxidase [HRP]) is added to the wells. The conjugate binds to antibodies, forming an antigen-antibody-HRP-conjugate complex. After washing to eliminate the excess conjugate, the substrate solution (3,3´,5,5´-tetramethylbenzidine) is added. In the presence of antibodies in the sample, a blue coloration appears, which becomes yellow after the addition of the stop solution. The intensity of coloration in each well depends on the quantity of specific antibodies present in the test sample. After the test, the microplates were read at 450 nm and the optical density of each solution was noted.

### Statistical analysis

Data from the laboratory analyses and surveys were entered into Microsoft^®^ Office Excel 2016 (Microsoft, USA). These data were then imported into R software to perform statistical analyses. The seroprevalence of infection was calculated by dividing the number of positive samples by the total number of samples collected per animal species. The Chi-square test or Fisher’s exact test was used to compare the variation of exposure to *Trichinella* by sex of animals, study area, and anatomical regions of sampling. For all statistical analyses, the significance level was set at 5%.

## Results

### Demographic characteristics of farmers

The socioeconomic data of farmers surveyed are mentioned in Table-1. Among interviewed people, 87.5% were from Dagana department and females (27.5%) were less involved in pigs breeding. Moreover, all farmers have never received training on pig breeding and the majority (62.5%) practiced pig farming for more than 10 years.

**Table-1:** Socio-professional data of pig farmers in the Saint-Louis region.

Variable	n	Percentage
Division		
Saint-Louis	5	12.5
Dagana	35	87.5
Sampling area		
Saint-Louis	5	12.5
Savoigne	15	37.5
Richard toll	20	50
Status		
Owner	40	100
Employee	0	0
Gender		
Female	11	27.5
Male	29	72.5
Breeding training		
Yes	0	0
No	40	100
Activity		
Farmer	7	17.5
Trader	5	12.5
Student	2	5
Public servant	7	17.5
Worker	11	27.5
No answer	8	20
Year of experience		
<5 years	3	7.5
Between 5 and 10 years	12	30
More than 10 years	25	62.5

### Type of farms and farm management

**Figure-1:**
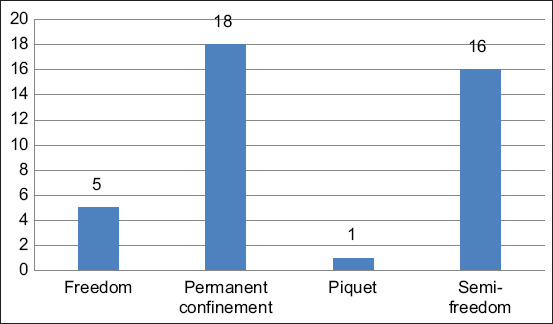
Breeding type relative to animal move restriction in Saint-Louis region, 2021.

**Figure-2:**
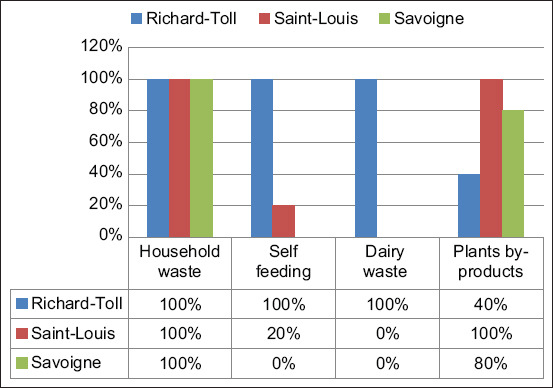
Food used for pigs breeding according to sampling area of Saint-Louis region, 2021.

**Figure-3:**
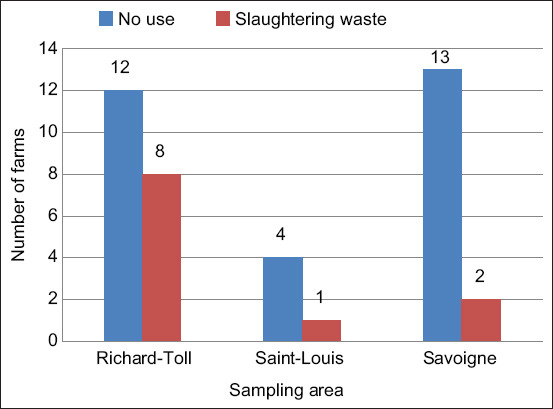
Proportion of farms using pig slaughter waste as pigs feed in Saint-Louis region, 2021.

The majority (97.5%) of the farms were extensive and farrow-to-finish. The construction of barns did not meet any standards and used makeshift materials such as leftover metal sheets, tree branches, and bricks. Among the farms visited, 40% were practicing free-ranging farming (Figure-1). Most farmers (97.5%) did not have the financial resources to manage their farms properly. Household waste was used to feed the pigs, and on some farms, the animals were fed with plant by-products (70%), swill from restaurants (50%), and wastes from dairies (50%), as shown in Figure-2. Dairy waste was only used in Richard Toll (Figure-2). In addition, farmers often let their animals run free to allow them to self-feed. However, some owners in the study area used wastes from slaughtered pigs to feed other pigs (Figure-3).

None of the farms applied biosecurity practices in farms. Hygiene on the farms was not acceptable. Animal health on the farms was not managed by a veterinarian and on some farms by a technician. There were no prophylaxis programs and animals were not dewormed. All interviewed farmers had never heard of trichinellosis. The slaughter of the animals was carried out by the farmers and the meat for human consumption was not inspected. They had no idea that animals could be infected with a pathogen if they were often left to roam freely and thus be a source of infection. The respondents rejected any possibility of a risk of transmission of certain diseases from pigs to humans and through the consumption of undercooked or uncooked pork.

### Seroprevalence and factors associated with *Trichinella* infection in pigs and warthogs in the northern area of Senegal

Among 201 pig samples tested (Table-2), 22 were positive, giving an overall seroprevalence of 10.9% (95% confidence interval [CI]: 6.6–15.2). The seroprevalence in pigs was significantly higher in males (22.2%, 95% CI: 6.6–37.8) than in females (9.2%, 95% CI: 4.9–13.5) (p < 0.05). However, there was no significant variation in the locations from where the samples were collected (p > 0.05). Samples taken from the pelvic limb had higher seroprevalence (56.3%) compared to other sampling sites in pigs, but this variation was not significant (p > 0.05).

**Table-2:** Seroprevalence and factors associated with *Trichinella* infection in pigs in Saint-Louis region, 2021.

Variables	n*	Positive	P (%) and 95% CI	p-value
Animal sex				
Males	27	6	22.2 (6.6–37.8)	0.04367
Females	174	16	9.2 (4.9–13.5)	
Sampling area				
Richard toll	79	11	13.9 (6.3–21.5	0.603
Ross-Bethio	7	0	-	
Saint-Louis	80	8	10.0 (3.5–16.5)	
Savoigne	35	3	8.6	
Anatomical area of sampling				
Abdomen	25	4	16.0 (1.7–30.3)	0.8394
Neck	11	0	0.0	
Pelvic limb	82	9	11.0 (4.3–17.7)	
Thoracic limb	16	2	12.5	
Thorax	47	5	10.6 (1.8–19.4)	
Head	20	2	10.0	

n*=Sample size for each modality, P= Seroprevalence, CI=The 95% confidence interval.

For warthogs, among 83 samples, nine were positive, with a seroprevalence of 10.8% (95% CI: 4.2–17.4). No significant variation has been noted according to animal sex, sampling area, and anatomical area of sampling (Table-3) (p > 0.05).

**Table-3:** Seroprevalence and factors associated with *Trichinella* infection in warthogs in Saint-Louis region, 2021.

Variables	n*	Positive	P (%) and CI	p-value
Animal sex				
Males	81	9	11.1 (4.3–17.9)	0.6176
Females	2	0	0.0	
Sampling area				
Diana	10	2	20.0	0.8141
Djoudj National Park	2	0	0.0	
Richard Toll	15	2	13.3	
Ross-Bethio	3	0	0,0	
Rosso	1	0	0,0	
Saint-Louis	23	1	4.3	
Savoigne	29	4	13.8	
Anatomical area of sampling				
Abdomen	12	1	8.3	0.2799
Neck	8	2	25.0	
Pelvic limb	11	1	9.1	
Thoracic limb	18	0	0.0	
Thorax	19	4	21.1	
Head	15	1	6.7	

n*=Sample size for the modality, P= Seroprevalence, CI=The 95% confidence interval

## Discussion

Saint-Louis is a region with three major pig production areas, led by Richard Toll, the main production center, followed by Savoigne and the city of Saint-Louis. Pig farming is mainly a male-dominated activity (72.5%). Indeed, this activity is often perceived as a male activity because it requires more effort and financial means. None of the farmers interviewed had received training in pig farming and the majority (63%) had more than 10 years of experience. This lack of training in pig farming could be justified by the fact that farmers consider this activity as a secondary activity and do not consider it necessary to have specific training in pig farming. The farms visited were of the traditional type and most farms (45%) confined the pigs permanently, 40% of the farmers kept the pigs semi-free-range and 12% of the farms kept the animals free-range. Free-ranging and semi-free-ranging animals can be a source of transmission of pathogens such as *Trichinella* as they often feed themselves and may ingest Trichinella-infested products or by-products [13]. In this study, the majority (97.5%) of the pig farmers interviewed were farrow-to-finish farmers and 2.5% were fatteners. Similar results were noted by Missohou *et al*. [14] in the Lower Casamance. However, regardless of the type (farrow-to-finish or fattener), the animals were most often fed with feed such as plant by-products (wheat bran, rice bran, maize bran, groundnut shells, etc.), dairy waste, and household waste without being treated beforehand. Indeed, farmers consider this type of farming more profitable than the fattening type because they have to buy the pigs before fattening them. However, the distribution of untreated swill that may contain *Trichinella*-infected meat could sustain the parasite cycle and ensure human infection [15].

It was also noted that farmers were slaughtering their animals themselves to sell the meat by the kilogram or the whole carcass. This problem is due to the absence of dedicated pig slaughterhouses in the Saint-Louis region. In Dakar, on the other hand, there is a slaughterhouse, but clandestine slaughter persists. In most localities, the absence of slaughterhouses means that pigs sold and consumed are slaughtered in households without any veterinary inspection. This uncontrolled mass slaughter of animals leads to disease transmission.

In Africa, the lack of means and the neglect of certain zoonoses mean that *Trichinella* is not systematically sought in slaughterhouses [16]. After having observed cases of human trichinellosis, it was necessary to understand the functioning of pig farms in the Saint-Louis region that could sustain the cycle of the parasite between pigs and wild animals. For this reason, the investigation of exposure to *Trichinella* in the muscles of pigs and warthogs intended for human consumption was necessary. The ELISA kit (ID Screen^®^ *Trichinella* Indirect Multi-species) available to us had the possibility to use plasma, serum, and meat juice. It was appropriate to use the meat juices from the muscle samples, as they were intended for human consumption.

In the present study, the seroprevalence of *Trichinella* infection from pig muscle samples was 10.9% compared to 10.8% obtained in warthogs. Serological studies implemented in pigs in Egypt, Nigeria, and Uganda reported seroprevalence of 4.5%, 40%, and 6.9%, respectively [17–19]. This difference in prevalence observed could be due to the management mode of the different farms and the hygiene applied. The existence of *Trichinella* infection in these countries reveals that the parasite could circulate in different countries of Africa. Some cases of *Trichinella* infection in warthogs have been reported in South Africa [20]. It was documented in 1973 by Vassiliades [5] with an overall prevalence of 4% in warthogs from Senegal and Mauritania. The higher seroprevalence found in our study reflects the negligence and insufficient epidemiological surveillance of this zoonosis, which could evolve if no measures are adopted to control it [21]. Seroprevalence in pigs was significantly higher in males (22.2%) than in females (9.2%). This difference could be linked to pigs’ management practices as pig owners prefer keeping males in the field than females. This could be a risk factor for *Trichinella* infection. In pig farms, owners opted for slaughtering females due to limited financial resources and limited space for breeding, as they could procreate at any time.

In the study area, most of the warthogs sampled were males, which is explained by the ban on hunting females in Senegal. The area of Savoigne was found to be the area with the highest seroprevalence. This locality is an important hunting area located near the Djoudj National Bird Park and a synergistic site, namely, the Diana hunting camp. The detection of anti-*Trichinella* antibodies in pigs and warthogs shows that the domestic and wild cycles are maintained. However, this cycle can be maintained by rats, which are reservoirs of the parasite [22, 23]. Cases of *Trichinella* infection have been reported in South Africa [20] and Egypt [17]. However, it is possible that rodents harbor the parasite in countries not yet studied, namely, Senegal. Thus, several cases of *Trichinella* infection in wild carnivores in Tunisia, Zimbabwe, South Africa, Egypt, Ethiopia, Guinea, Mozambique, Namibia, DR Congo, Senegal, and Tanzania show that carnivores are also a reservoir for the parasite [17, 20, 24–27].

Control of these rodents, hygienic measures on farms, adequate feeding, keeping pigs in permanent confinement, and effective farm management are practical ways to control the maintenance cycle of the parasite. Therefore, surveillance of other wild animals, such as carnivores, is important for better control of the disease.

## Conclusion

This study determined the circulation of *Trichinella* in pig and warthog meat in the Saint-Louis region. The existence of the parasite in the muscle indicates that there is a possibility of transmission to humans. The inadequate management of pig farms in this region could sustain the parasite cycle and constitute risk factors. As trichinellosis is a major zoonosis, it is important to communicate about its existence and to prohibit poor farm management. However, it is advisable to cook Suidae meat thoroughly before eating it.

## Authors’ Contributions

OBG, LDD, KMN, and AT: Designed the research. KMN and NESB: Performed the field activities. KMN and NESB: Analyzed the data and interpretation. JK and AT: Participated in the development of the discussion and the correction of the document in English. All authors have read and approved the final manuscript.
